# Ti_3_C_2_T_X_ MXene/Polyaniline-Modified Nylon Fabric Electrode for Wearable Non-Invasive Glucose Monitoring in Sweat

**DOI:** 10.3390/bios15080531

**Published:** 2025-08-14

**Authors:** Lichao Wang, Meng Li, Shengnan Ya, Hang Tian, Kerui Li, Qinghong Zhang, Yaogang Li, Hongzhi Wang, Chengyi Hou

**Affiliations:** 1 State Key Laboratory of Advanced Fiber Materials, College of Materials Science and Engineering, Donghua University, Shanghai 201620, China; meng.li2@mt.com (M.L.); 1169145@mail.dhu.edu.cn (H.T.); likr@dhu.edu.cn (K.L.); zhangqh@dhu.edu.cn (Q.Z.); yaogang_li@dhu.edu.cn (Y.L.); hcy@dhu.edu.cn (C.H.); 2 School of Medical Imageology, Wannan Medical College, Wuhu 241002, China; snya@wnmc.edu.cn; 3 Mettler Toledo, Shanghai 200233, China

**Keywords:** non-invasive, nylon fabrics, sweat glucose detection, Ti_3_C_2_T_x_ MXene/polyaniline, electrochemical sensor

## Abstract

Sweat-based electrochemical sensors for wearable applications have attracted substantial interest due to their non-invasive nature, compact design, and ability to provide real-time data. Remarkable advancements have been made in integrating these devices into flexible platforms. While thin-film polymer substrates are frequently employed for their durability, the prolonged buildup of sweat on such materials can disrupt consistent sensing performance and adversely affect skin comfort over extended periods. Therefore, investigating lightweight, comfortable, and breathable base materials for constructing working electrodes is essential for producing flexible and breathable sweat electrochemical sensors. In this study, nylon fabric was chosen as the base material for constructing the working electrode. The electrode is prepared using a straightforward printing process, incorporating Ti_3_C_2_T_X_ MXene/polyaniline and methylene blue as modification materials in the electronic intermediary layer. The synergistic effect of the modified layer and the multi-level structure of the current collector enhances the electrochemical kinetics on the electrode surface, improves electron transmission efficiency, and enables the nylon fabric-based electrode to accurately and selectively measure glucose concentration in sweat. It exhibits a wide linear range (0.04~3.08 mM), high sensitivity (3.11 μA·mM^−1^), strong anti-interference capabilities, and high stability. This system can monitor glucose levels and trends in sweat, facilitating the assessment of daily sugar intake for personal health management.

## 1. Introduction

Wearable and portable sensors for real-time, continuous, in vitro health monitoring have experienced explosive growth in recent years [1,2,3,4,5]. Electrochemical detection is the most widely used method in wearable sweat sensors [6,7,8,9,10]. The substrate of the working electrode plays a crucial role in the sensing performance of wearable sweat electrochemical sensors. Most wearable sweat detection devices are currently integrated on substrates like plastic (PET), polyimide, polyurethane, polydimethylsiloxane, and polymethyl methacrylate [11]. Excessive sweat accumulation on high-strength polymer substrates hinders continuous device analysis and long-term skin comfort. Efficient sweat processing that ensures comfort while maintaining high sensitivity and stability has become a new requirement. Moreover, the closed working electrode’s contact with human skin interferes with sweat gland secretion and disrupts heat dissipation balance [12]. Prolonged wear of such biosensors may cause skin inflammation and itching. These challenges hinder the advancement of accurate sweat component detection and continuous health monitoring. Therefore, it is essential to explore lighter, more comfortable, and breathable substrate materials to construct working electrodes for flexible and breathable sweat biosensors. Advances in smart fiber textile and flexible electronics technologies have led to textile-based wearable sweat sensors offering innovative solutions for real-time diabetes monitoring [13,14]. Compared to polymer films and paper, textiles offer adjustable three-dimensional porous structures, enhanced air and moisture permeability, large surface areas, and strong mechanical flexibility. They can withstand bending, twisting, shearing, and stretching. Their diverse fiber and weaving patterns, low cost, and excellent biocompatibility provide more substrate options for wearable sweat sensors [15,16]. In recent years, materials inspired by natural wetting behaviors have received considerable interest for their capability to facilitate controlled liquid handling on demand. The strategic design of surfaces with asymmetric characteristics—such as geometry, texture, or surface energy—has propelled the advancement of unidirectional fluid transport systems. In particular, Janus wettable structures, comprising two closely adhered layers with contrasting wettability, have emerged as an effective solution for directional moisture removal and the promotion of dry surface detachment [17,18,19]. Most can degrade harmlessly after disposal. Textile-based wearable sweat sensors fit comfortably on human skin without restricting movement [20,21]. They detect sweat biomarkers for non-invasive, real-time health monitoring by integrating with electrode-modified sensitive materials.

In electrochemical detection, sensitive materials are key components of the sensor’s working electrode [22,23]. Currently, various strategies are utilized to enhance the analytical performance of electrochemical sweat sensors. Among these, designing and regulating the composition and structure of sensitive materials is an effective method to enhance sensor performance [24,25,26]. New two-dimensional nanomaterials with high specific surface area, electrochemical activity, and conductivity are gaining increasing attention in this field [27,28,29]. For instance, Ti_3_C_2_T_X_, a representative of the MXenes family, exhibits excellent enzyme immobilization performance, good biocompatibility, and a high specific surface area, making it promising for sensing applications [30,31,32,33]. However, the hydrophilic nature of MXenes end groups (e.g., -OH, -O, or -F) results in poor stability due to the hydration–oxidation process in humid environments, potentially altering interfacial electron transport and causing potential drift. This phenomenon affects the application of MXenes in electrochemical sensors [34]. To address these issues, designing new high-performance MXene-based hybrids and developing simple, cost-effective preparation methods are essential. Polyaniline (PANI), a conductive polymer, is commonly used in electrochemical sensors, batteries, supercapacitors, and other applications due to its environmental stability, abundant adsorption sites, and large specific surface area [35,36]. Conductive composites with an extensive electroactive surface facilitate charge transfer processes at the electrode–electrolyte interface, increase the available active area for reactions, and aid in the stable immobilization of biomolecules, thereby enhancing the overall detection capabilities of electrochemical sensing systems [37,38]. Recent studies have shown that the combination of MXene and polyaniline shows potential applications in biosensors, energy, etc. For example, Zhao et al. used polyaniline/MXene (PANI/Ti_3_C_2_T_X_) nanocomposites to improve the electrocatalytic sensitivity of gas sensors [39]. A. VahidMohammadi et al. synthesized MXene/PANI hybrid electrodes with good capacitance retention by in situ oxidant-free polymerization of PANI on the surface of 2D MXene nanosheets [40].

In this study, we systematically evaluated the feasibility of various textile materials as electrode substrates for wearable sweat sensors, ultimately selecting nylon fabric as the optimal substrate for constructing flexible electrodes in electrochemical sensor assembly. Ti_3_C_2_T_X_/PANI nanocomposites were synthesized using low-temperature in situ polymerization to ensure high-quality polyaniline polymerization while minimizing damage to Ti_3_C_2_T_X_. The Ti_3_C_2_T_X_/PANI nanocomposites were applied to modify the working electrode, incorporating methylene blue (MB) as an electron mediator to enhance glucose detection performance in sweat. Glucose oxidase was then immobilized to develop an electrochemical sweat glucose sensor with targeted recognition capabilities. The performance of the modified electrode and sensor was further characterized. Ti_3_C_2_T_X_/PANI composite nanomaterials exhibit high conductivity and biocompatibility, while the macromolecular structure of PANI facilitates enzyme immobilization on the electrode surface. The synergistic combination of MB and Ti_3_C_2_T_X_/PANI enhances electron transfer at the electrode surface, thus improving sensor sensitivity and stability. This study also investigates the sensor’s concentration–current relationship, anti-interference properties, and long-term stability. The glucose sensor demonstrates a sensitivity of 3.11 μA·mM^−1^ and a detection limit of 4.82 μM within a concentration range of 0.04~3.08 mM. The results indicate that the nylon-based wearable electrochemical sweat sensor effectively leverages the fabric’s flexibility, breathability, and stable sensing performance, demonstrating promising potential for new wearable sensors aimed at monitoring physiological parameters.

## 2. Materials and Methods

### 2.1. Synthesis of Ti_3_C_2_T_X_ Dispersion

As reported in previous studies, Ti_3_C_2_T_X_ was synthesized through etching with hydrofluoric acid followed by lithium chloride intercalation [41]. Initially, 1 g of Ti_3_AlC_2_ powder was gradually introduced into a mixed solution containing 2 mL of hydrogen fluoride, 12 mL of 9 M hydrochloric acid, and 6 mL of ultrapure water. The mixture was maintained at 25 °C under continuous magnetic stirring for 24 h. After the reaction, the resulting suspension was subjected to repeated washing with ultrapure water. Following each centrifugation step, the supernatant was discarded until a neutral pH of approximately 6 was achieved, indicating completion of the washing process. Add the washed Ti_3_C_2_T_X_ to the mixed solution of LiCl (1 g) and ultrapure water (100 mL) and stir it magnetically for 1 h. The purpose of adding LiCl in this step is to allow small molecules to enter between the multilayer Ti_3_C_2_T_X_ layers, and through intercalation, the interlayers of the multilayer Ti_3_C_2_T_X_ layers are better stretched to form single-layer Ti_3_C_2_T_X_ nanosheets. Subsequently, the stirred mixed solution was centrifuged and washed with ultrapure water at 3500 rpm for 1 h to remove lithium-ion salts. The Ti_3_C_2_T_X_ solution was vigorously shaken by hand to ensure dispersion while retaining the large lamellar structure of Ti_3_C_2_T_X_. Finally, the supernatant was collected by centrifugation at 3500 rpm for 1 min, and the supernatant was a large monolayer suspension of Ti_3_C_2_T_X_ nanomaterials. After the above process, a large monolayer Ti_3_C_2_T_X_ suspension was prepared at a concentration of 5.0 mg/mL by dilution with ultrapure water and stored at 4 °C for subsequent experiments.

### 2.2. Synthesis of Ti_3_C_2_T_X_/PANI Nanocomposites

The synthesis procedure for Ti_3_C_2_T_X_/PANI nanocomposites was conducted according to the following experimental steps: First, 10 mL of high-concentration Ti_3_C_2_T_X_ (20 mg/mL) dispersion was added to 20 mL of HCl solution (1 M). Subsequently, 100 μL of aniline (ANI) was added to the above mixed solution, and magnetically stirred at 5 °C for 30 min. Meanwhile, 0.228 g ammonium persulfate (APS) powder was dispersed in 10 mL of 1 M HCl solution to prepare the oxidation. After 30 min, the APS solution was gradually introduced into the homogeneously mixed Ti_3_C_2_T_X_/ANI solution. Subsequently, the mixture was stirred magnetically for 6 h at a temperature between 0 °C and 5 °C. The resulting suspension was centrifuged and washed three times with ethanol and deionized water, yielding the Ti_3_C_2_T_X_/PANI composite powder. The resulting powder was subsequently dried under vacuum at room temperature for 5 h.

### 2.3. Electrochemical Measurement

Cyclic voltammetry (CV) and chronoamperometry (amperometric i–t curves) were conducted using a three-electrode system with an electrochemical potentiostat (CHI 760D, CH Instruments Inc., Shanghai, China). Electrochemical impedance spectroscopy (EIS) measurements were performed on an electrochemical workstation (Bio-Logic SAS, VSP-300 with EC-Lab software, version 10.40). EIS, based on AC impedance, was carried out in a 0.1 M KCl solution containing 5 mM [Fe(CN)_6_]^3−^/^4−^. Amperometric i–t curves were recorded at −0.18 V in analyte solutions of varying concentrations to characterize glucose. Unless otherwise specified, all measurements were conducted at room temperature. To evaluate sensor stability, performance was monitored over a 15-day period, and the samples were stored at 4 °C to preserve the enzymatic activity on the sensor.

## 3. Results

### 3.1. Characterization of Ti_3_C_2_T_X_/PANI Electrode Modification Materials

**Figure 1:**
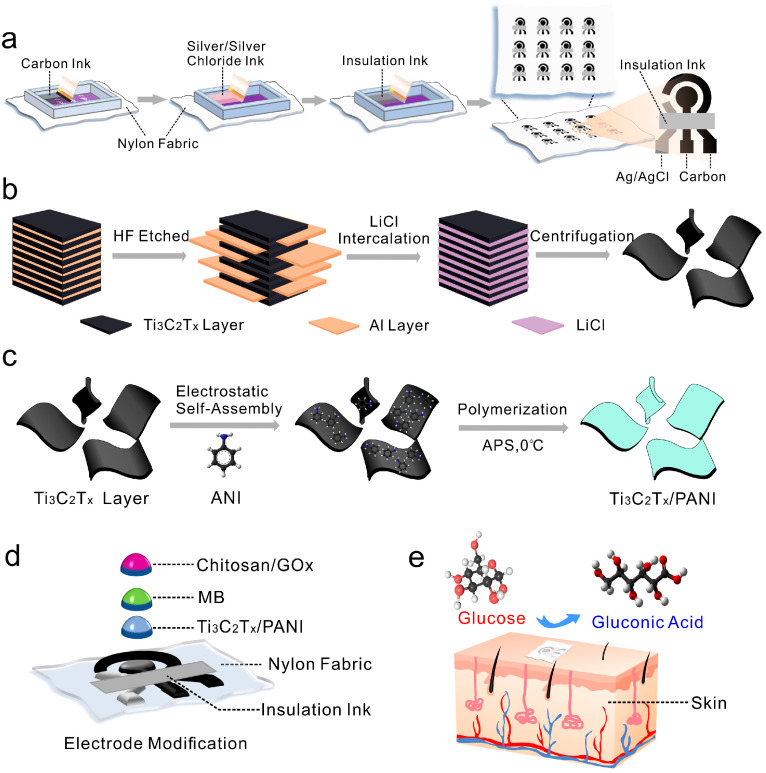
Schematic diagram of (**a**) nylon fabric-based electrochemical sweat sensor electrode prepared by screen printing process. The formation of Ti_3_C_2_T_X_/PANI nanocomposite includes (**b**) the stripping process of Ti_3_AlC_2_ and (**c**) the polymerization process of ANI. Schematic illustration of (**d**) electrochemical sensor electrode structure based on nylon fabric and (**e**) non-invasive wearable sweat biosensor for sweat glucose detection using Ti_3_C_2_T_X_/PANI nanocomposite layer.

The procedure for fabricating electrodes via a screen-printing process on nylon fabric selected as the substrate is shown in Figure 1a, with additional synthesis details available in the Supporting Information (SI). By constructing basic carbon electrodes on nylon, pure cotton, linen, cupro, and polyester fabrics, for example, Figure 1a illustrates the selection of nylon fabrics as the substrate, and the use of a low-cost, batch-preparable screen-printing process to fabricate carbon current collector electrodes. The specific preparation process and related experimental results were discussed in the SI. It is found that compared with other fabric electrodes, the nylon electrode has a larger crystalline area within the macromolecule, a smaller amorphous area and a compact molecular structure [42]. It is difficult for water molecules to enter the interior of the macromolecule, and the contact angle (Figure S1) of sweat on its surface will not change significantly within a certain period of time. This ensures sufficient contact time between sweat and the target analytes, thereby improving the stability of the measurements. Ag/AgCl paste and carbon paste were uniformly applied onto the surface of the nylon fabric substrate (Figure S2). The electrode surface of the cotton fabric is loose and porous in the microscopic state, which affects the conductivity of the electrode. Cotton-based electrodes exhibited a more pronounced change in resistance after the washing test (Figure S3), indicating poor suitability for long-term and repeated use. In contrast, nylon fabric demonstrated better suitability for constructing this type of sweat glucose electrode.

Pure cotton and nylon-based fabric electrodes were selected to characterize their elemental states. Additional information regarding the binding energy between the electrodes and the substrate was obtained from the valence states of the elements (Figure S4). In the flexible fabric electrode modified by screen printing, the valence state of C 1s changed, enabling a strong attachment of the electrode printing material to the fabric substrate. Compared to pure cotton fabric, nylon-based fabric has more chemical bonds, which enhances the stability of the electrode and ensures reliability for subsequent tests. As shown in Figure 1b,c, the exfoliation of Ti_3_AlC_2_ and the low-temperature in situ polymerization used to synthesize Ti_3_C_2_T_X_/PANI nanocomposites are illustrated. The specific preparation procedures for Ti_3_C_2_T_X_ nanosheets and Ti_3_C_2_T_X_/PANI composites are provided in the SI. A small amount of Ti_3_C_2_T_X_/PANI/Nafion mixed solution was drop-cast onto the surface of the carbon electrode. After drying, specific substances such as methylene blue and the enzyme solution were added dropwise to achieve the configuration shown in Figure 1d. Sweat is rich in physiological biomarkers and provides insight into an individual’s current health status. Figure 1e illustrates the structure of sweat glands and the working mechanism of a non-invasive, wearable sweat biosensor integrated with a flexible, fabric-based sensing interface.

**Figure 2:**
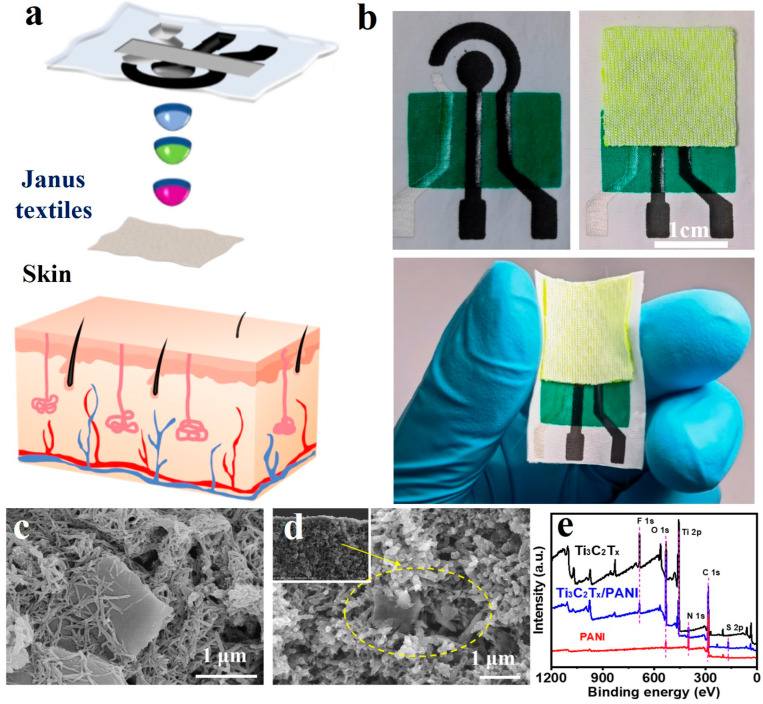
(**a**) Three-dimensional exploded schematic illustrating the design of the integrated smart nylon fabric for autonomous sweat sampling and electrochemical detection, highlighting the sequential subsystems of the flexible hybrid sensor from top to bottom. (**b**) Picture of the nylon-based electrochemical sensing fabric and the composite of Janus fabric and nylon sensing fabric. The FESEM image of (**c**) Ti_3_C_2_T_X_/PANI nanocomposite and (**d**) localized cross-section. (**e**) XPS survey spectra of Ti_3_C_2_T_X_, PANI, and Ti_3_C_2_T_X_/PANI.

Figure 2 illustrates the overall design strategy of a wearable sweat electrochemical sensor based on Janus textiles, in which nylon fabric is employed for both sweat collection and biosensing. As shown in Figure 2a, the sensor’s system-level structure comprises four sequential layers, arranged from top to bottom. First is an economical nylon fabric that facilitates attachment to human skin. Second, electrochemical sensing is achieved through electrodes prepared via screen printing and subsequent chemical modification. Third, an insulating ink layer serves as a dielectric barrier, preventing sweat from contacting the carbon electrode pathways and thereby ensuring stable electrical signal output. Lastly, the fourth layer consists of a Janus textile that is directly integrated into the nylon substrate and serves as a critical component for enhancing sample collection and directional sweat transport. This functional textile exhibits asymmetric wettability, characterized by a hydrophilic outer surface and a hydrophobic inner surface, thereby facilitating unidirectional sweat transport from the skin to the sensing interface and effectively inhibiting reverse diffusion. The integration of the Janus layer into the nylon base provides enhanced mechanical stability, intimate skin conformity, and reduced signal interference during movement. Moreover, this design enables sustained capillary-driven sweat transport, forming a passive microfluidic network that maintains stable sample delivery to the sensing region, thereby improving the reliability and responsiveness of the wearable biosensor. Figure 2b demonstrates that the sensing electrode exhibits high flexibility, allowing it to closely adhere to the skin and effectively minimize the interface gap between the device and the human body. This enables immediate acquisition of fresh sweat and prevents the loss of target analytes, thereby ensuring the accuracy of real-time analysis.

As depicted in Figure S5, the textile featuring distinctive Janus wettability not only efficiently captures and directs sweat in a single direction but also securely holds it at the electrode interface. This retention initiates a series of electrochemical reactions, producing a current signal proportional to the analyte concentration. When the nylon-based sweat sensor is worn on the skin, the hydrophobic side of the Janus textile faces the epidermis, while the hydrophilic side contacts the electrode. Sweat continuously secreted by the subcutaneous glands passes through the hydrophobic layer and accumulates on the hydrophilic surface. As shown in Figures S6–S8, the characterization results for MXene, PANI, and Ti_3_C_2_T_X_/PANI, along with detailed discussions, are provided in the SI. Figure 2c shows the surface morphology of Ti_3_C_2_T_X_/PANI nanocomposites prepared using low-temperature in situ polymerization. From Figure 2d, it can be clearly observed that PANI is coated on the surface of Ti_3_C_2_T_X_ lamellar structure, indicating that Ti_3_C_2_T_X_/PANI nanocomposites were successfully synthesized. The chemical structures of the prepared materials were further analyzed using X-ray photoelectron spectroscopy (XPS). Figure 2e illustrates the XPS spectra of PANI, Ti_3_C_2_T_X_, and Ti_3_C_2_T_X_/PANI nanocomposites. The presence of titanium (Ti), carbon (C), oxygen (O), and fluorine (F) elements is clearly evident in both materials. The XPS pattern of the Ti_3_C_2_T_X_/PANI nanocomposites demonstrated the presence of nitrogen (N) compared to the Ti_3_C_2_T_X_MXene (Figure S8), thereby providing further evidence for the successful synthesis of the Ti_3_C_2_T_X_/PANI nanocomposites.

### 3.2. Electrochemical Performance Testing of Bare Electrodes for Fabric-Based Sensors

**Figure 3:**
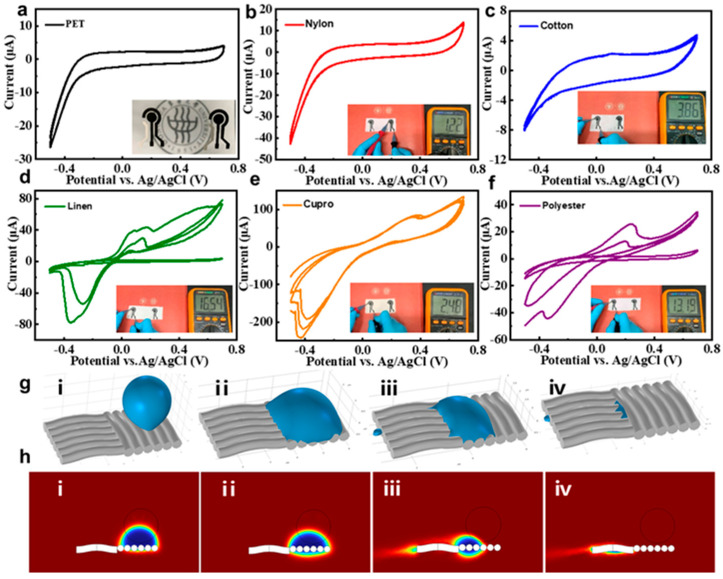
CV curves of (**a**) PET, (**b**) nylon, (**c**) cotton, (**d**) linen, (**e**) cupro, and (**f**) polyester substrate sensor electrodes in PBS solution. (**g**,**h**) Penetration and diffusion behavior of droplets on nylon surface simulated by COMSOL Multiphysics software (version 6.3). (**g**): (**i**–**iv**): 3D droplet evolution on the structured surface, from initial contact to full spreading and partial detachment. (**h**): (**i**–**iv**): 2D cross-sections showing droplet profile, spreading, flow development, and final shape.

Electrochemical sensors on various fabric substrates were fabricated using a screen-printing process, and their electrochemical stability was evaluated via CV. Figure 3a shows the electrochemical sensors prepared on PET material (used as a control), which exhibited stable electrochemical properties. The CV curves of sensors on nylon and cotton substrates (Figure 3b,c) displayed no significant current variation or peak potential drift, with the nylon-based sensors closely resembling the PET-based ones. Both nylon and cotton substrates demonstrated superior electrochemical stability. Additionally, as depicted in Figure S1a,b, nylon showed good stability over one minute. In contrast, Figure 3d,e highlight significant signal drift during the second and third CV scan cycles, consistent with hydrophilicity test results in Figure S1. The extreme hydrophilicity of linen, copper-ammonia, and polyester fabrics caused rapid liquid diffusion across the fabric surface, preventing the working electrode from achieving a stable signal. Furthermore, the insets in Figure 3b–f illustrate the resistance of different fabric substrate electrodes, corresponding to the magnitude of the CV current signals. Substrates with relatively dense weaves, such as cotton, nylon, and copper–ammonia, exhibited better electrical conductivity. However, due to its high hydrophilicity, the copper–ammonia fabric failed to sustain stable signal transmission.

As shown in Figure S3, cotton-based electrodes underwent notable resistance changes after ultrasonic washing, making them unsuitable for long-term reuse. Nylon, on the other hand, exhibited superior performance. Nylon fabric forms stronger covalent bonds with carbon ink, resulting in minimal peak potential shift and negligible impedance change after repeated CV tests and ultrasonic washing. Therefore, nylon was chosen as the substrate material for the screen-printed fabric-based sweat glucose sensing electrode.

Nylon’s unique properties contribute to its effectiveness in sweat sensing. As shown in Figure S1, nylon is relatively hydrophobic, promoting sweat aggregation on the fabric surface. Nylon’s molecular structure, containing nitrogen atoms, features both hydrophobic (carbonyl and alkyl groups) and hydrophilic (amide bonds and amino groups) components. This dual nature allows limited hydrogen bonding with water molecules, resulting in moderate hydrophilicity. Additionally, nylon’s compact molecular structure, characterized by a large crystalline region and small amorphous region, slows water penetration and ensures a stable contact angle for sweat over time [43].

The screen-printed carbon electrode exhibits inherent hydrophilicity, allowing for rapid sweat absorption. Utilizing Janus wettable material, water gathers on the hydrophilic side and partially contacts the nylon-based sweat sensing electrode, extending the interaction time between sweat and target analytes. This configuration enhances enzymatic reaction efficiency and ensures stable electrochemical signal output. Theoretical calculations based on the experimental results suggest that the moisture–fabric interface can be approximated as a capillary model with a wettability gradient. This gradient facilitates rapid droplet penetration into the carbon material’s inner layers. Fluid simulations of nylon fabric using COMSOL Multiphysics software (version 6.3), (Figure 3g,h) confirmed these observations, indicating that the composite structure of carbon material and nylon fabric can effectively conduct water. This property is expected to support subsequent electrochemical reactions while maintaining a comfortable microenvironment for the skin.

Biocompatibility testing further validated the suitability of the nylon-based sensors. Figure S9 presents fluorescence images of cells cultured with electrochemical sensing fabrics. After 3 days of incubation at 37 °C and 5% CO_2_, cell viability remained high at 85%, with relatively few dead cells, demonstrating the excellent biocompatibility of the nylon-based electrochemical sensing fabric. Moreover, no visible signs of skin irritation or inflammation were observed after 24 h of continuous electrode application (Figure S10).

### 3.3. Electrochemical Performance Characterization of Ti_3_C_2_T_X_/PANI Modified Electrode

**Figure 4:**
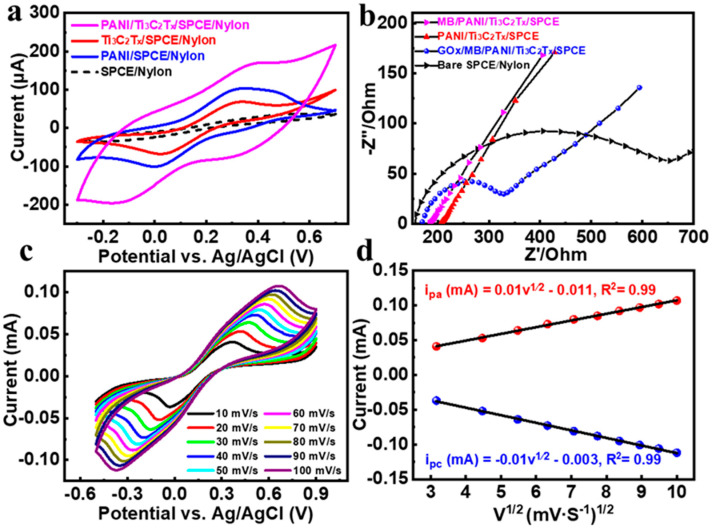
(**a**) CV curves of Ti_3_C_2_T_X_, PANI, and Ti_3_C_2_T_X_/PANI material modified SPCEs in a 0.1 M PBS solution at a scan rate of 20 mV/s. (**b**) EIS for a series of electrodes in a 0.1 M KCl solution containing 5.0 mM [Fe(CN)_6_]^3−^/^4−^. (**c**) The CV response of Ti_3_C_2_T_X_/PANI electrode-modified materials in 5.0 mM [Fe(CN)_6_]^3−/4−^ containing 0.1 M KCl solution at different scan rates. (**d**) Plot of measured redox peak current versus the square root of the scan rate (ν^1/2^).

On the basis of nylon fabric, enzyme-based amperometric sensors for glucose were designed and fabricated (see Figure S11 for the working mechanism of glucose sensor). The electrochemical property changes in the modified materials of the glucose sensor electrode modified layer by layer by drop-coating method were characterized by electrochemical techniques. As shown in Figure 4a, the nylon-based sensor electrodes were modified with Ti_3_C_2_T_X_, PANI, and Ti_3_C_2_T_X_/PANI materials and tested for CV, respectively. When Ti_3_C_2_T_X_/PANI nanocomposites were modified on the working electrodes, the area of the CV curves and peak currents were significantly increased, and the sensors showed higher current signals and achieved a true promotion of the current responses’ synergistic effect. The EIS signal changes in Figure 4b further demonstrate the significant potentiation of electrochemical signals by the presence of MB and Ti_3_C_2_T_X_/PANI mixtures. In addition, the modification with two bioenzymes reduced the electron transfer efficiency of the electrode, indicating the successful immobilization of the bioenzymes on the electrode surface. The CV curves of MB/Ti_3_C_2_T_X_/PANI/screen-printed carbon electrodes (SPCEs) in the probe solution at scan rates ranging from 10 to 100 mV/s are shown in Figure 4c. The peak current and the curve area increased with the scan rate, demonstrating a highly reversible redox process. It indicates that the glucose oxidase (GOx)/MB/Ti_3_C_2_T_X_/PANI electrode modification material possesses good electron transfer ability and electrochemical stability. As shown in Figure 4d, a linear correlation between the redox peak current and the square root of the sweep (i~v^1/2^) was presented and described by the following equations: *i_pa_* (mA) = 0.01v^1⁄2^ − 0.011 (R^2^ = 0.99); *i_pc_* (mA) = −0.01v^1⁄2^ − 0.003 (R^2^ = 0.99).

### 3.4. Characterization of Glucose Sensing Performance

**Figure 5:**
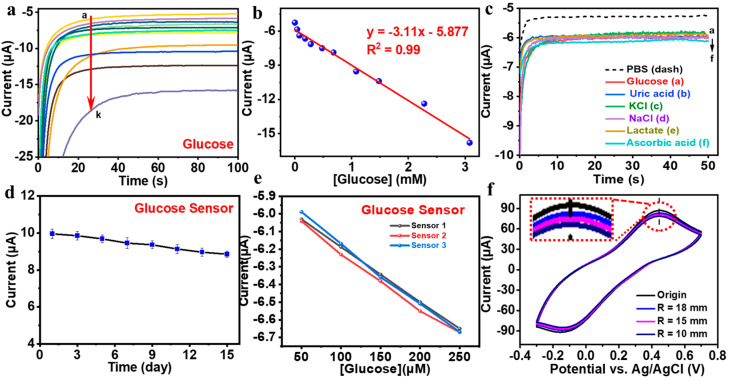
Current response (**a**) and calibration curve (**b**) of nylon-based electrochemical sweat sensor with curves a–k representing glucose concentrations of 0, 0.04, 0.08, 0.18, 0.28, 0.48, 0.68, 1.08, 1.48, 2.28, and 3.08 mM, respectively. (**c**) The current–time curve of nylon-based sweat glucose electrochemical sensor tested in 0.15 mM glucose solution and 1 mM lactic acid, uric acid, ascorbic acid, NaCl, and KCl interference solution (containing 0.15 mM glucose). (**d**) Cycle stability of nylon-based sweat glucose electrochemical sensor in 1.5 mM glucose solution. (**e**) Reproducibility of glucose sensors produced in different batches (Sensor 1, Sensor 2, Sensor 3) at different concentrations of glucose. (**f**) The CV signal of nylon-based sweat glucose electrochemical sensor under different bending radii.

To further assess the electrochemical detection capabilities of the nylon-based electrochemical sweat sensor, the relationship between its steady-state current and glucose concentration was analyzed. As depicted in Figure 5a, the data were analyzed using linear regression, which revealed a linear correlation between current and glucose concentration. In Figure 5b, within the glucose concentration range of 0.04 to 3.08 mM, the fitted equation is expressed as y = −3.11x −5.877 (R^2^ = 0.99). The glucose sensor’s current response stabilizes over time, with an increase in current corresponding to higher glucose concentrations. The linear fit indicates a sensitivity of 3.11 μA·mM^−1^ and a detection limit of 4.82 μM for this concentration range. These findings confirm that the glucose sensor’s sensitivity and linearity are appropriate for measuring glucose levels in real sweat. Compared with previously reported screen-printed electrochemical glucose sensors, the fabricated electrode demonstrates markedly enhanced catalytic activity, as reflected in its higher sensitivity, lower detection limit, and wider linear detection range for glucose detection (Table S1). These performance improvements are primarily attributed to the synergistic integration of Ti_3_C_2_T_X_ MXene and polyaniline, which together afford a large electroactive surface area, excellent electrical conductivity, and a high density of active sites for glucose oxidation. To evaluate the accuracy of the wearable sensor in dynamic sweat monitoring, a control experiment was conducted to compare sweat glucose measurements obtained from the wearable sensor with those from commercial device. The results from the glucose assay kit were consistent with those of the sweat sensor (Figure S12), demonstrating the satisfactory accuracy of the sensor in analyzing real sweat samples.

To enhance the practical applicability of this sensor, it is crucial to evaluate its selectivity, long-term stability, and repeatability, especially for nylon fabric-based wearable sensors. As shown in Figure 5c, the working electrode of the sweat sensor recorded the current–time response in a glucose solution containing fixed concentrations of ascorbic acid, lactic acid, uric acid, NaCl, and KCl. The steady-state currents remained stable at the same potential, and no significant interference signals were observed. It shows that the sweat sensor is highly specific for glucose biomarkers, which is beneficial to the accurate detection of sweat glucose content. Take multiple nylon-based electrochemical sweat sensors to test their periodic stability and record the ampere response current of the sensors in the liquid to be measured at the same concentration at time intervals. As shown in Figure 5d, the sensor exhibited minimal degradation in current response over a 14-day storage period, indicating sustained electrochemical stability over time. This result further confirms its robust operational stability under extended storage conditions and its suitability for integration into long-term wearable systems for continuous glucose monitoring. Figure 5e demonstrates that glucose sensors fabricated from different batches exhibited comparable responses, indicating reliable reproducibility. As shown in Figure 5f and Figure S13 bending stability is a critical requirement for wearable sensors, and the results further confirm the mechanical stability of the nylon-based sweat glucose sensor. In static bending tests, the electrode oxidation peak currents showed a slight decrease, while no notable shifts in oxidation potential were observed. Even when the bending radius was reduced to 10 mm, neither the oxidation potential nor the peak current exhibited significant changes. These results demonstrate that the nylon-based sweat glucose sensor exhibits strong electrochemical stability under mechanical deformation.

## 4. Conclusions

In summary, we evaluated the feasibility of different fabric materials for wearable sweat sensors by comparing their properties. Based on the comparison, nylon fabric was chosen as the substrate for the electrochemical sweat sensor. Ti_3_C_2_T_X_/PANI material was synthesized and modified on the electrode surface, in combination with an MB electronic mediator, to synergistically enhance electron transfer efficiency. Immobilizing glucose oxidase on the electrode produced an electrochemical sweat glucose sensor with specific recognition capabilities. Characterization of the modified electrode materials and sensor performance showed that Ti_3_C_2_T_X_/PANI composite nanomaterials exhibit excellent electrical conductivity and biocompatibility. The macromolecular structure of PANI also facilitates enzyme immobilization on the electrode surface. The Ti_3_C_2_T_X_/PANI composite enhances electron transport on the electrode surface, further improving sensor sensitivity. The concentration–current relationship, anti-interference capability, and long-term stability of the sensor were also evaluated. The glucose sensor showed a sensitivity of 3.11 μA·mM^−1^ and a detection limit of 4.82 μM within the glucose concentration range of 0.04~3.08 mM. Testing in artificial sweat confirmed its robust performance and stability under simulated conditions. The results indicate that the nylon-based wearable electrochemical sweat sensor benefits from the flexibility, breathability, and stability of its fabric substrate. This sensor holds promising application potential for monitoring physiological parameters in wearable devices.

## Data Availability

The original contributions presented in this study are included in the article/Supplementary Materials. Further inquiries can be directed to the corresponding author (L.W.).

## Supplementary Materials

The following supporting information can be downloaded at: https://www.mdpi.com/article/10.3390/bios15080531/s1, Figure S1: Contact angles of (a) nylon, (b) cotton, (c) linen, (d) cupro, and (e) polyester fabrics after 0 sec and 1 min, where the (d,e) plots show photographs of the fabrics’ contact angles taken after 1 s. (H = hight, D = diameter). Figure S2: FESEM images of silver/silver chloride reference electrodes on cotton (a) and nylon (c) fabric substrates, and carbon working electrodes on (b) cotton and (d) nylon fabric substrates. Figure S3: Resistance changes in pure cotton and nylon-based electrodes under stirring, ultrasonic washing and heating conditions. Figure S4: XPS spectrum of fabric and fabric/SPCE surface (a) XPS spectrum of pure cotton and cotton/SPCE, (b) element spectrum of pure cotton C1s, (c) element spectrum of cotton/SPCE C1s, (d) XPS spectrum of pure nylon and nylon /SPCE, (e) element spectrum of pure nylon C1s, (f) element spectrum of nylon/SPCE C1s. Figure S5: (a,b) One-way moisture absorption of Janus textiles. Water droplets penetrate from the hydrophobic surface and spread on the hydrophilic side. Figure S6: FESEM images of (a) PANI and (b) Ti_3_C_2_T_X_. Figure S7: XRD patterns of Ti_3_C_2_T_X_, PANI and Ti_3_C_2_T_X_/PANI. Figure S8: High-resolution XPS spectra of (a) Ti 2p, (b) C 1s, (c) O 1s, and (d) N 1s for Ti_3_C_2_T_X_/PANI. Figure S9: Fluorescence images of live and dead cells after cell viability test using CCK-8 assay. Figure S10: Photographs of the forearm taken before (a), during (b), and after (c) 24 h of wearing the Janus textile integrated with the Ti_3_C_2_T_X_ MXene/polyaniline-modified nylon fabric electrode, affixed using medical adhesive tape. Figure S11: Schematic diagram showing the working mechanisms of glucose sensor. Figure S12: Detection of glucose concentration in sweat samples by a self-made glucose sensor and glucose assay kit, respectively. Figure S13: The ratio of the current (I/I_0_) obtained from CV signals of the nylon-based sweat glucose electrochemical sensor after 100 bending cycles under various bending radii, where I and I_0_ represent the peak oxidation current after and before bending, respectively. Figure S14: Storage stability of the biosensor in artificial sweat ([glucose]—100 µM). Table S1: Comparison of the performance of the fabricated screen-printed electrochemical glucose sensor with other reported ones. Refs. [44,45,46,47,48,49,50,51,52,53,54,55,56,57,58,59,60,61,62,63,64,65] cited in Supplementary Materials.

## Abbreviations

The following abbreviations are used in this manuscript:

SPCE	Screen-printed carbon electrodes
GOx	Glucose oxidase
MB	Methylene blue
APS	Ammonium persulfate
ANI	Aniline
PANI	Polyaniline
CV	Cyclic voltammetry
EIS	Electrochemical impedance spectroscopy
