# Supplementary material for: Ti3C2TX MXene/Polyaniline-Modified Nylon Fabric Electrode for Wearable Non-Invasive Glucose Monitoring in Sweat
Source: Biosensors (Basel). 2025 Aug 14;15(8):531. doi: 10.3390/bios15080531 (PMC12385094; doi:10.3390/bios15080531)
Supplement: Supplementary file 1 [file biosensors-15-00531-s001.zip › biosensors-3783819-supplementary.pdf]

Supplementary Material

$Ti_3C_2T_X$ MXene/Polyaniline-Modified Nylon Fabric Electrode for Wearable Non-Invasive Glucose Monitoring in Sweat

Lichao Wang [1,2,†,*], Meng Li [1,3,†], Shengnan Ya [2], Hang Tian [1], Kerui Li [1], Qinghong Zhang [1], Yaogang Li [1], Hongzhi Wang [1,*] and Chengyi Hou [1]

[1] State Key Laboratory of Advanced Fiber Materials, College of Materials Science and Engineering, Donghua University, Shanghai 201620, China; 1169145@mail.dhu.edu.cn (H.T.); likr@dhu.edu.cn (K.L.); zhangqh@dhu.edu.cn (Q.Z.); yaogang_li@dhu.edu.cn (Y.L.); hcy@dhu.edu.cn (C.H.)

[2] School of Medical Imageology, Wannan Medical College, Wuhu 241002, China; snya@wnmc.edu.cn

[3] Mettler Toledo Shanghai, 200233, China; meng.li2@mt.com

† These authors contributed equally to this work.

* Correspondence: wlch168@wnmc.edu.cn (L.W.); wanghz@dhu.edu.cn (H.W.)

1. Experimental Section

1.1. Chemicals and reagents

Titanium aluminum carbide (Ti_3AlC_2, 98 %, 200 mesh) was purchased from Jilin 11 technology Co., Ltd (China). Hydrofluoric acid (HF, 50 wt%) was obtained from Shanghai Aladdin Biochemical Technology Co., Ltd. Aniline (ANI, C_6H_7N, 99.5 %) was provided from Bailingwei Technology Co., Ltd. Chitosan, ammonium persulphate (APS, $(NH_4)_2S_2O_8$, AR), lactate ($C_3H_6O_3$, AR), lithium chloride (LiCl, AR, 99 %) and hydrochloric acid (HCl, 36 wt%) were supplied by Sinopharm Chemical Reagent Co., Ltd. Nafion-117 (5.0 wt%, AR) and phosphate-buffer solution (PBS, KH_2PO_4 and K_2HPO_4) and ascorbic acid ($C_6H_8O_6$, ≥ 99.0 %) were obtained from Sigma Aldrich Chemicals Co. Ltd (USA). Glucose ($C_6H_{12}O_6$, ≥ 99.0 %) and methylene blue (MB, $C_{16}H_{18}ClN_3S$, AR) were purchased from Shanghai Macklin Biochemical Co. Ltd. Potassium ferricyanide ($K_3[Fe(CN)_6]$, AR), Potassium hexacyanoferrate (II) ($K_4[Fe(CN)_6]$, ≥ 99.0 %), Uric acid ($C_5H_4N_4O_3$, ≥ 99.0 %), potassium chloride (KCl, ≥ 99.0 %), and sodium chloride (NaCl, ≥ 99.0 %) were purchased from the Alfa Aesar (China) Chemical Co., Ltd. Glucose oxidase (GOx, S10020-50ku, 250 u/mg) was purchased from Shanghai Yingsuo Biotechnology Centre (China). Different fabrics (nylon, cotton, linen, cupro, polyester), Janus textile (Quick-wicking fabric) and

polyethylene glycol terephthalate (PET) were purchased from Taobao e-commerce platform (China). Artificial sweat was obtained from Guangzhou Deli Chemical (China) Co., Ltd. The screen-printed carbon electrodes (SPCE) were made from carbon ink and silver/silver chloride ink, with two carbon electrodes acting as the working electrode (WE) and counter electrode (CE), respectively. The Ag/AgCl electrode was made from silver/silver chloride ink as a reference electrode (RE). Both carbon and silver/silver chloride inks were purchased from Shenzhen D-MAX Technology Co. Ltd. (Shenzhen, China). Insulation ink was purchased from Shitiao Printing Equipment Technology (Pinghu) Co., Ltd, China. D Milli-Q water with 18.2 Ω cm^{-1} was generated in the water purifier. (Pinching Technology Co., Ltd.) and used throughout the experiment. These chemicals were used without further purification.

1.2. Sample characterization

X-ray diffraction (XRD) analysis was carried out on a Bruker D8 Discover (Germany) X-ray diffractometer with a Cu Kα X-ray source (λ = 1.5418 Å). Field emission scanning electron microscopy (FESEM) images were conducted using Hitachi S-4800 with 5 kV under high vacuum conditions. Characterization of MXene/polyaniline was completed by transmission electron microscopy (Talos F200S, FEI NanoPorts, USA). The compositions and chemical states of samples were

confirmed by X-ray photoelectron spectroscopy (XPS) using a Theta Probe (Escalab 250Xi, Thermo Fisher Scientific, USA). The contact angle of fabrics was characterized using a contact angle analyzer (OCA40Micro, Germany). In addition, the resistance variation was measured by the digital multimeter (VICTOR VC890C+, Victory Instrument Equipment Co., Ltd., China). Glucose content in sweat was quantified using an enzyme-labeled instrument (TECAN Infinite F50, Switzerland) and assessed with a glucose content assay kit (Shenggong Bioengineering (Shanghai, China) Co., Ltd).

1.3. Fabrication of nylon fabric-based screen-printed electrodes

A three-electrode pattern suitable for sweat detection on human surfaces was designed using Adobe Illustration software, and a screen-printing plate for screen printing was customized from the pattern. The fabric-based electrochemical sweat sensor electrodes were fabricated as follows. Appropriately sized fabrics (nylon, linen, cupro-ammonia, polyester, and cotton materials) were placed under an appropriate number of screen-printing plates. Highly conductive carbon paste (as working electrode, counter electrode, respectively) and silver/silver chloride paste (as reference electrode) were screen-printed on it in batches and then dried in an oven at 70 °C for 2 minutes. Ultimately, various fabric electrodes were obtained.

1.4. Comparison and selection of fabric-based materials

1.4.1 Electrochemical performance comparison of fabric-based sensor electrodes

In a comparative test of the electrochemical properties of different fabric substrates, the electrode stability of the fabric substrates was tested using CV with electrochemical workstation (CHI 760D). PBS solution (80 μL, pH = 7.2) was dropped on the surface of bare electrodes based on different fabrics for CV test. The initial voltage was -0.5 V, the final voltage was 0.7 V, and the scanning was performed 3 times at a scanning speed of 20 mV/s. According to the CV curves, the electrochemical stability of each fabric base was observed. Electrochemical impedance spectroscopy measurements were carried out in a 0.1 M KCl solution containing 5.0 mM $[Fe(CN)_6]^{3-/4-}$ over a frequency range of 100 mHz to 2 MHz, utilizing a Bio-logic SAS VSP-300 workstation. Unless otherwise stated, all reported potentials are referenced against the Ag/AgCl electrode.

1.4.2. Electrode stability comparison of fabric-based sensors

Based on the electrochemical performance results of various fabric-based sensor electrodes, two substrates with good electrochemical stability were selected to compare the physical stability of the electrodes and to further

explore their feasibility for applications in wearable electrochemical sensors. The specific experiments included treating the screen-printed electrodes, based on the two stable substrate fabrics, with vigorous stirring in water, ultrasonic cleaning, and oven heating. Changes in electrode resistance over time under different conditions were then observed. Cleaning and stirring were conducted at a magnetic stirrer speed of 1500 rpm and an ultrasonic power of 200 W. Heating experiments were conducted in an oven at 60°C.

1.5. Modification of nylon-based electrochemical sweat sensor electrodes

The working electrode of the nylon-based electrochemical sweat sensor was modified using a layer-by-layer drop-coating method, as detailed below. First, the nylon-based working electrode's surface was rinsed with 100 mM PBS buffer (pH = 7.2) and dried in an oven at 30°C. A 20 μL $Ti_3C_2T_x$/PANI dispersion (0.5 mg mL^{-1}, containing 0.1% Nafion) was evenly applied to the electrode surface using a microsampler and dried at 10°C to obtain $Ti_3C_2T_x$/PANI/SPCEs. Next, 10 μL of 5 mM MB solution was added dropwise to the surface of the $Ti_3C_2T_x$/PANI/SPCEs using a microsampler, dried at room temperature, and stored at 4°C for 3 h. Finally, a glucose oxidase solution (60 mg mL^{-1}, containing 5% chitosan and 2% acetic acid) was applied to the surface of the

$MB/Ti_3C_2T_x/PANI/SPCEs$, dried at room temperature, and stored at 4°C for future use.

1.6. Electrochemical performance test of nylon-based electrochemical sweat sensor

1.6.1. Sensing performance test of nylon-based electrochemical sweat sensor

In the sensing performance test, the current-time (i-t) method of the electrochemical workstation was used to test the relationship between glucose concentration and current, and a standard concentration curve was drawn based on the concentration–current relationship. First, glucose standard solutions of 1 mM, 2.5 mM, 5 mM, 10 mM and 20 mM were first prepared using 100 mM PBS buffer solution (phosphate–buffer solution was used for the preparation of subsequent solutions). First, 50 μL of PBS buffer solution was placed on the surface of the three electrodes and scanned using the i-t method at an operating voltage of -0.18 V until the baseline was stabilized (approximately 100 seconds). The solution was removed and 50 μL of PBS buffer solution was again taken on the electrode surface and scanned at the same voltage for 50 s as a signal from the blank substrate. Glucose standard solution (2 μL) was then gradually added to the fabric electrode surface to increase the

concentration gradient of glucose test solution (0, 0.04, 0.08, 0.18, 0.28, 0.48, 0.68, 1.08, 1.48, 2.28 and 3.08 mM). For each addition, the solution was pipetted with a micro-syringe for 15 seconds to allow glucose oxidase to fully catalyze and oxidize the solution to be tested. The i-t curve was recorded after each concentration change and the glucose concentration-current relationship was fitted.

1.6.2. Anti-interference performance test of nylon-based electrochemical sweat sensor

In the anti-interference performance test of the nylon-based electrochemical sweat sensor, 50 μL of PBS buffer solution was applied to the electrode surface. The baseline was stabilized using the i-t method at an operating voltage of -0.18 V for approximately 100 seconds. Then, the current-time curve was recorded, and histograms were generated using a glucose standard solution (0.15 mM) and its interference solutions. The interference solutions consisted of 1 mM lactic acid, uric acid, ascorbic acid, NaCl and KCl, each containing 0.15 mM glucose.

1.6.3. Long-term stability test of nylon-based electrochemical sweat sensor

A batch of nylon electrochemical sweat sensors was prepared and stored in a sealed refrigerator at 4°C. The sensors were removed daily and

subjected to i-t scanning in a 2 mM glucose standard solution. Additionally, every two days, the sensors were also scanned in the same solution, and the steady-state current values were recorded each time.

1.6.4. On-body evaluation of sweat Glucose.

On-body assessment of sweat glucose was conducted. During in situ monitoring, a wearable sensor integrated with the Janus textile was attached to the subject's arm. To induce sufficient sweating, the subject ran continuously for ten minutes. Electrochemical signals were then recorded using chronoamperometry. Sweat samples were analyzed using commercial device, including an enzyme-labeled instrument and a glucose content assay kit. Sweat samples for ex situ analysis were collected using capillary tubes from the same skin site as the on-body measurement, and the volunteer’s arm was disinfected with alcohol after each sampling. The applicability of the electrochemical glucose sensor was validated by comparing sweat glucose concentrations measured by the sensor with those obtained from standard analytical methods.

Results and Discussion

Selection and analysis of fabric-based materials

Hydrophilicity characterization of fabric-based materials

It is essential for sweat to remain on the sensor surface briefly, when selecting substrates for electrochemical sweat sensors. This ensures that the biomarkers in sweat react with the surface-modifying enzymes on the working electrodes to detect chemical signals. Consequently, nylon, cotton, linen, cupro, and polyester fabrics were characterized for their hydrophilic properties to evaluate changes in contact angle over a brief period. Nylon fabrics are synthetic fibers characterized by lipophilic organic groups within the macromolecular chain. These fabrics lack polar groups, such as -OH, -NH, and C=O, that form hydrogen bonds with water molecules. Furthermore, nylon has a relatively large crystalline area and a small amorphous region, resulting in a compact molecular structure. This makes it difficult for water molecules to penetrate the fiber's internal space. As shown in **Figure S1a, b**, nylon demonstrates good stability within one minute. The contact angle of cotton also changes minimally during this time. Both fabrics exhibit characteristics suitable for electrochemical sweat sensor substrates. As shown in **Figure S1c-e**, linen, cupro, and polyester fabrics diffuse rapidly upon contact with water droplets, either after one minute or one second. This rapid diffusion prevents sufficient contact between sweat and the working electrode surface, failing to meet the requirements for an electrochemical sweat sensor substrate.

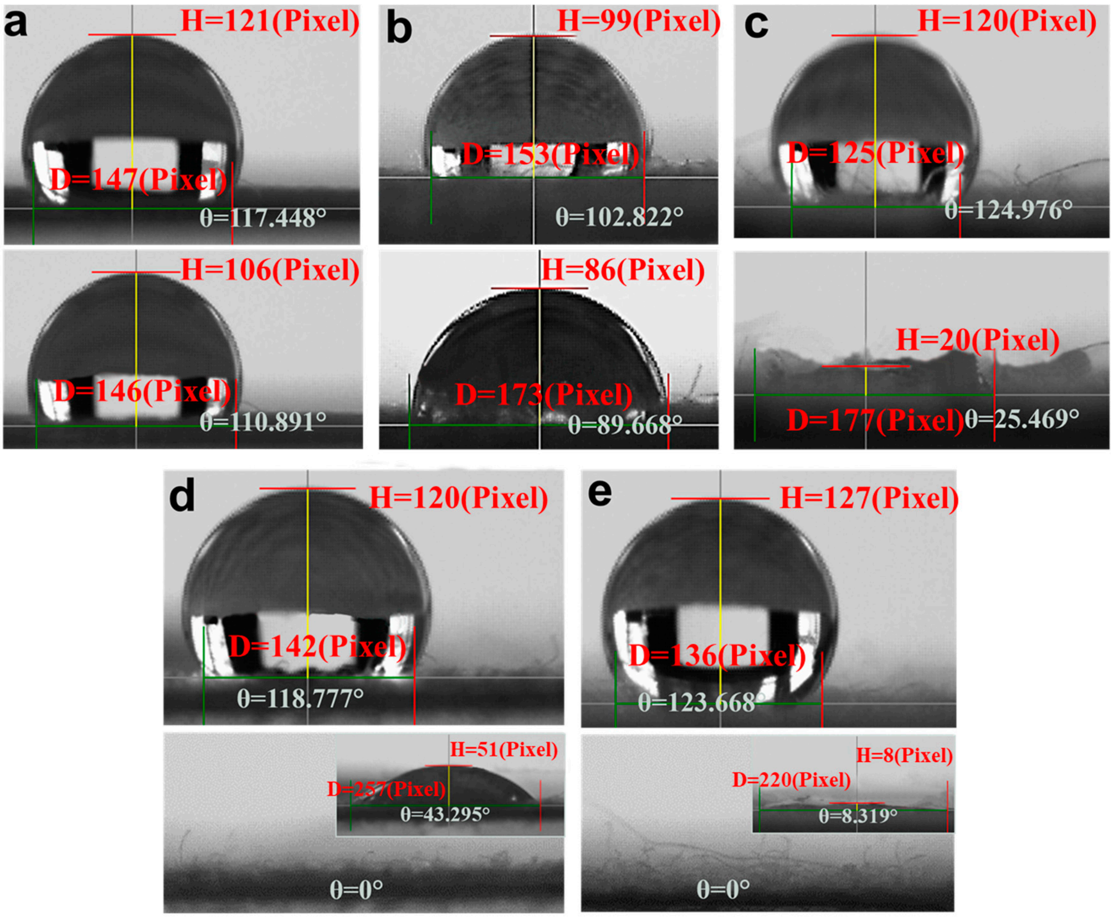

Figure S1. Contact angles of (a) nylon, (b) cotton, (c) linen, (d) cupro, and (e) polyester fabrics after 0 sec and 1 min, where the (d, e) plots show photographs of the fabrics' contact angles taken after 1 sec. (H = height, D = diameter).

Morphological characterization of bare electrodes on fabric surfaces

The surface morphology of the bare electrodes of the cotton and nylon-based sensors was observed by field emission scanning electron microscopy. As shown in **Figure S2**, it can be observed that Ag/AgCl paste and carbon paste have been uniformly coated on the nylon fabric substrate surface by screen printing process. However, cotton fabric

cannot achieve the same dense effect as nylon fabric in terms of weave count, which causes the surface of cotton fabric electrode to be loose and porous at the microscopic level, affecting the conductivity of the electrode.

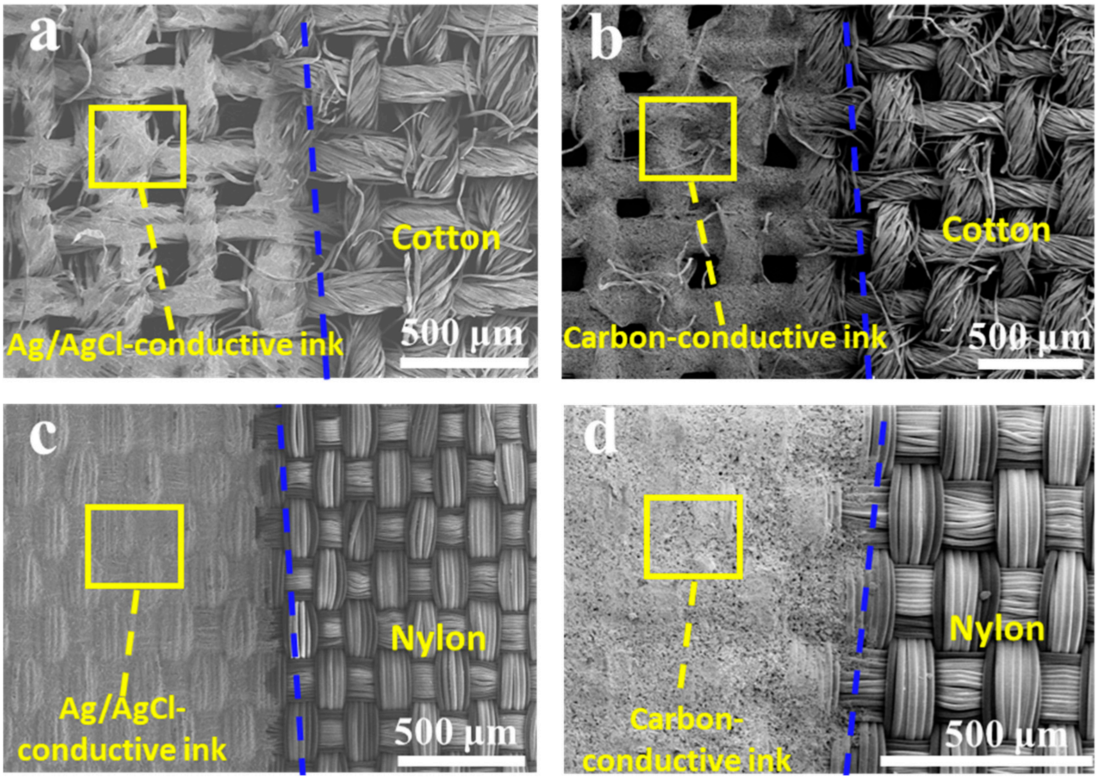

Figure S2. FESEM images of silver/silver chloride reference electrodes on cotton (a) and nylon (c) fabric substrates, and carbon working electrodes on (b) cotton and (d) nylon fabric substrates.

Stability testing of bare electrodes for pure cotton and nylon-based sensors

The resistance changes in cotton and nylon fabrics were tested under different conditions so as to compare the physical stability of fabric-based

electrodes. As shown in **Figure S3**, the electrode resistance of the cotton substrate increased significantly after 90 minutes of agitation during vigorous agitation in water, and the nylon-based electrode resistance changed less with time. During ultrasonic water washing, the electrode resistance of the cotton substrate was more affected, and the electrode resistance of the nylon-based had a small change after 150 min of ultrasonication. Under heating conditions, the electrode resistances of the two fabric substrates changed less, indicating that the effect of temperature on the electrode properties was not significant, while ultrasound had a significant effect on the fabric electrodes. After washing test, the cotton-based electrode resistance change is more obvious, which is not suitable for long-term reuse requirements; relatively speaking, it is more suitable for constructing this kind of sweat glucose electrode on nylon fabric. There is more covalent bonding with carbon ink, the peak potential shift is not obvious after many CV tests, and the electrode impedance change is not significant after many ultrasonic washings, which is more suitable as the substrate material of the carbon-based electrode. Therefore, nylon fabric was used as the substrate material, and ink was used as the collector material to prepare the collector layer of the screen-printed fabric-based sweat glucose sensing electrode in this experiment.

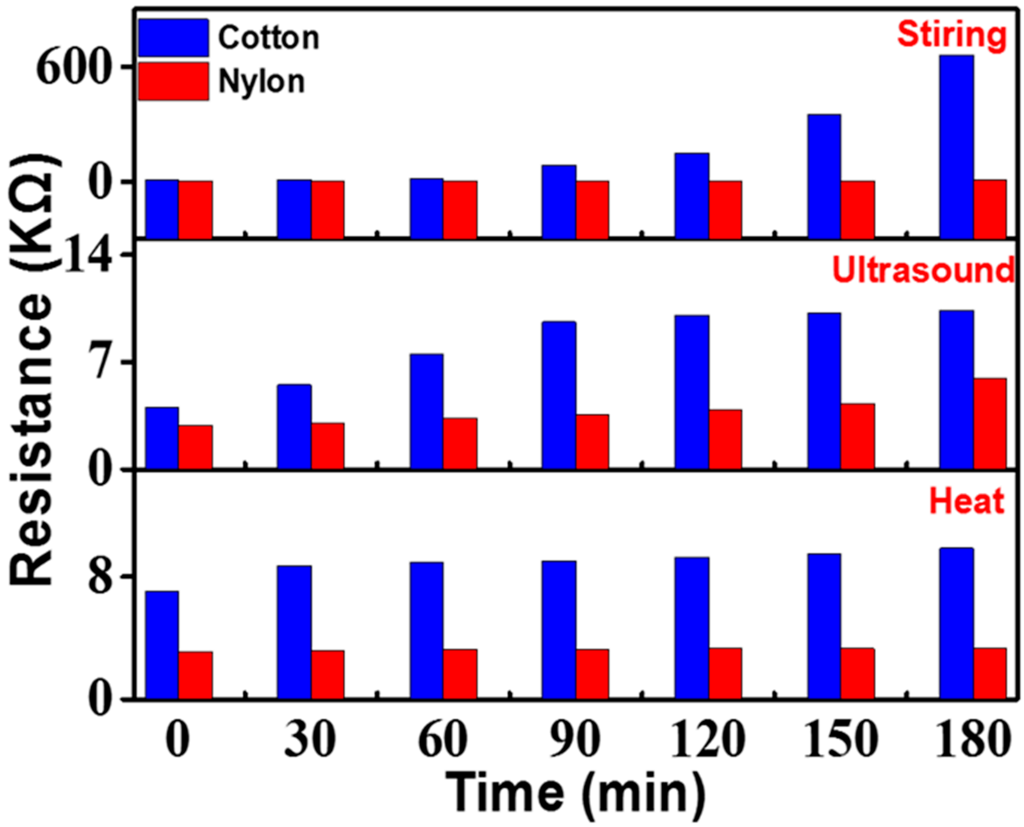

Figure S3. Resistance changes in pure cotton and nylon-based electrodes under stirring, ultrasonic washing and heating conditions.

XPS characterization of bare electrodes on pure cotton, nylon and their fabric surfaces

As can be seen from **Figure S4a, d**, since cotton is mainly composed of cellulose fibers and nylon is polyester, both substrates before and after the printed electrodes are enriched with a large number of C and O elements, as well as a small number of impurity signal peaks containing Si. By etching the fabric samples with printed electrodes for 1 min and collecting the elemental signals, it can be seen that the fabric-based electrode samples have more Cl 2p characteristic peaks, which come

from the additives in the carbon paste used for the printed electrodes. As shown in **Figure S4b, c**, the following phenomenon can be observed in the C 1s narrow spectrograms of the untreated cotton fabric and the fabric-based electrodes: for the untreated cotton substrate, there are three characteristic peaks at the positions of 284.8, 286.4, and 286.9 eV, which correspond to the C-C bond, the C-O bond, and the C=O bond, respectively[44] (**Figure S4b**). After screen-printed carbon paste, the intensity of the signal peak of C-O bond was significantly enhanced, and the half-height width of the signal peak of C=O bond was reduced and the intensity increased (**Figure S4c**), which indicated that the conductive carbon paste printed on the surface of the cotton and the fiber did not just simply have a physical adhesion effect, but also had a certain chemical bond binding effect, which led to an increase in the bonding of the active conductive material with the fabric substrate. As shown in **Figure S4e, f**, the C 1s fractional peak fitting of nylon and its nylon-based electrodes is different from that of pure cotton fabrics. First, there is no C=O bonding signal peak in the pure nylon fabric, but a new peak position attributed to O-C=O bonding appears at 291.9 eV[45]; second, the intensity of the O-C=O bonding signal peak on the surface of the modified electrodes is weakened, while the signal peak of C=O bonding appears at 288.7 eV, which proves that the multiple chemical bonding interactions also existed

between nylon and its printed electrodes. The above analysis fully proves that the valence state of C 1s is changed in the screen printed modified flexible fabric electrode so that the electrode printed material is closely adhered to the fabric substrate.

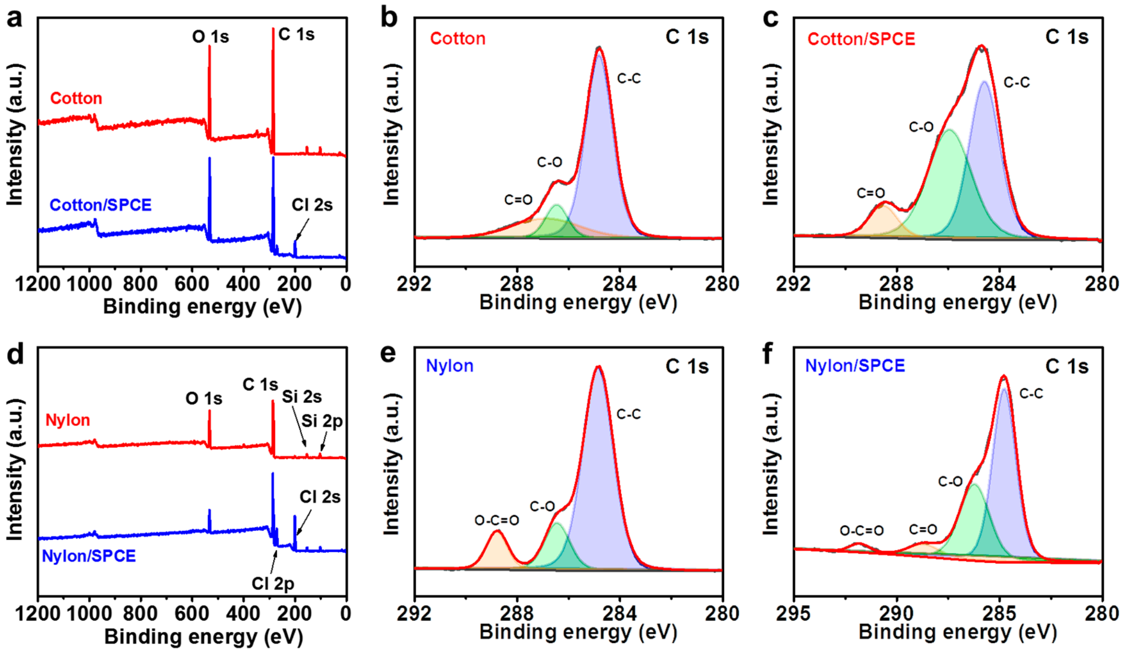

Figure S4. XPS spectrum of fabric and fabric/SPCE surface (a) XPS spectrum of pure cotton and cotton/SPCE, (b) element spectrum of pure cotton C1s, (c) element spectrum of cotton/SPCE C1s, (d) XPS spectrum of pure nylon and nylon /SPCE, (e) element spectrum of pure nylon C1s, (f) element spectrum of nylon/SPCE C1s.

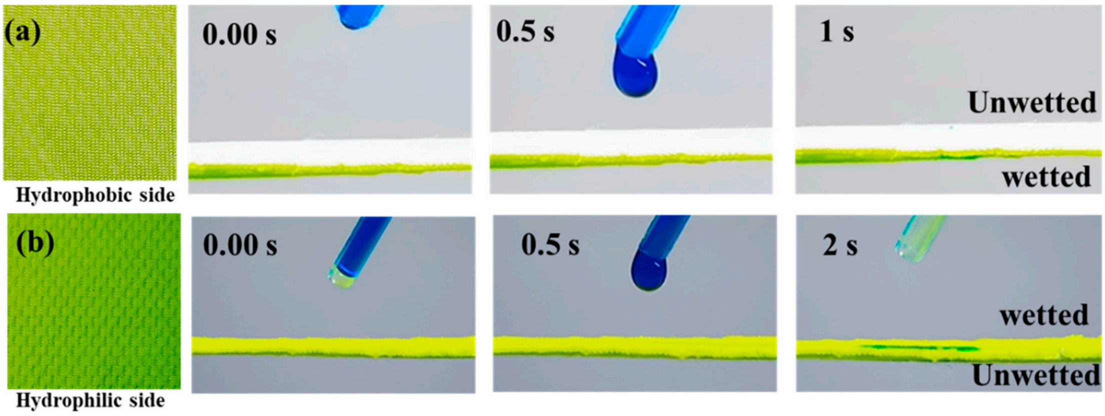

Figure S5. (a, b) One-way moisture absorption of Janus textiles. Water droplets penetrate from the hydrophobic surface and spread on the hydrophilic side.

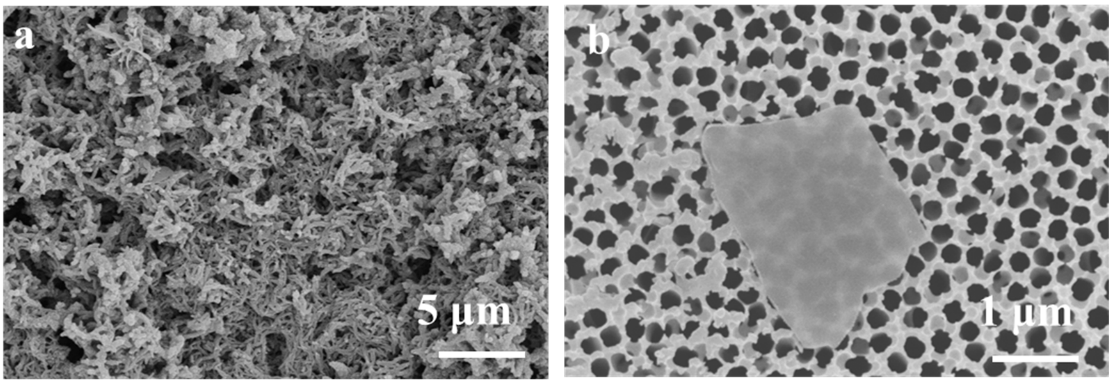

Figure S6. FESEM images of (a) PANI and (b) Ti_3C_2Tx.

Figure S6 shows the FESEM images of conducting polyaniline and Ti_3C_2Tx nanosheets. As can be seen in **Figure S6a**, the synthesized conductive polymer PANI mainly exhibits a nanorod-like morphology, which clearly demonstrates the reliability of the chemical oxidation method employed to generate PANI through the polymerization reaction of ANI monomers. In addition, the highly conductive PANI exhibits a dark green color on the macroscopic scale, and its electrochemical

performance is significantly better than that of intrinsically black PANI. As shown in **Figure S6b**, Ti_3AlC_2 has been successfully etched and layered into $Ti_3C_2T_x$ nanosheets, which exhibits an obvious layer structure and the prepared nanosheets are very thin and transparent, which well illustrates the 2D properties of MXene.

To further investigate the composition and structure of $Ti_3C_2T_x$/PANI, the compositional components of PANI, $Ti_3C_2T_x$MXene, and $Ti_3C_2T_x$ /PANI nanocomposites were analyzed using XRD spectroscopy. **Figure S7** presents the XRD spectra of the two-dimensional layered materials: $Ti_3C_2T_x$, PANI, and $Ti_3C_2T_x$/PANI. A series of characteristic periodic peaks of $Ti_3C_2T_x$ appear at $2\theta = 7.1°$, 13.1°, 19.8°, and 26.7°, corresponding to the (002), (004), (006), and (008) crystal faces[46]. A strong (002) peak appears at $2\theta = 7.1°$, with a calculated d-spacing of 6.21 Å based on the Bragg formula. Pure PANI exhibits a characteristic crystallization peak at $2\theta = 14.9°$, representing a regular periodic signal in the polyaniline molecular chain, consistent with the literature report[47]. $Ti_3C_2T_x$/PANI composite nanomaterials exhibit characteristic peaks at 15.1°, 20.5°, and 25.6°, attributed to the layered structure introduced by $Ti_3C_2T_x$ and the crystallization peaks of polyaniline. Additionally, because PANI is coated on the surface of $Ti_3C_2T_x$, its amorphous structure broadens the full width at half maximum of each peak. In

summary, the XRD characterization results confirm the successful compounding of the material based on its phase structure.

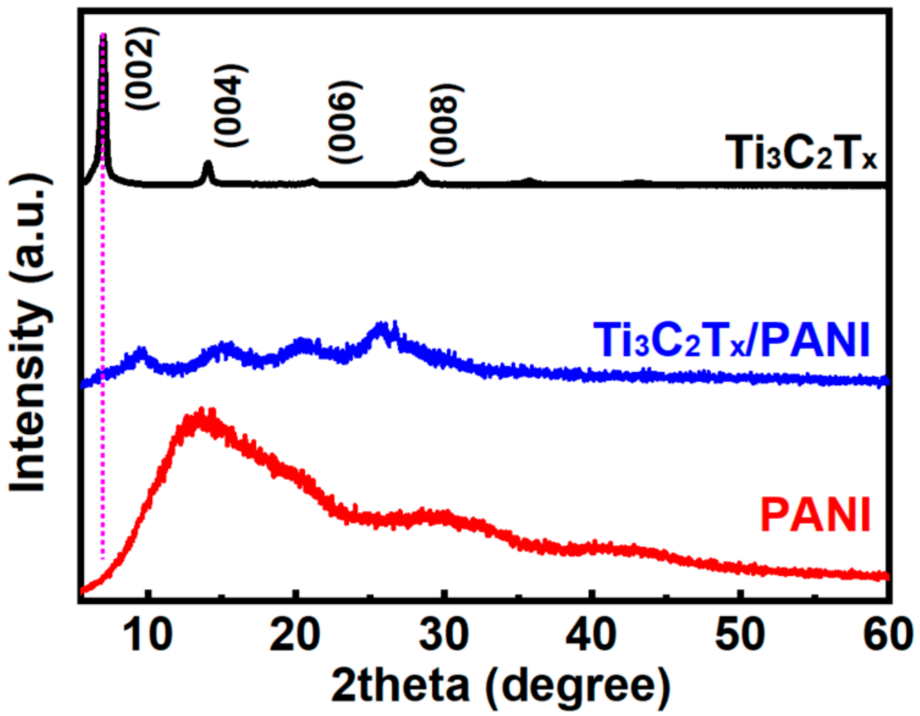

Figure S7. XRD patterns of $Ti_3C_2T_x$, PANI and $Ti_3C_2T_x$/PANI.

The Ti 2p spectra (**Figure S8a**) exhibited typical peaks at binding energies of 454.9 eV, 461.1 eV, 455.6 eV, 461.2 eV, 456.82 eV, and 462.0 eV, which are in good agreement with previous reports and are assigned to Ti-C, Ti(II)-O, and Ti(III)-O, respectively. Additionally, the peaks observed at binding energies of 458.5 eV and 463.2 eV, as well as 459.6 eV and 464.3 eV, confirm the binding of Ti(IV)-O and Ti(IV)-F[48], respectively. The C 1s spectrum (**Figure S8b**) exhibits three peaks at 282.0 eV, 284.8 eV, 286.6 eV, and 288.8 eV, corresponding to Ti-C, C-C, C-N, and C=O[49], respectively. As illustrated in **Figure S8c**, the O 1s

spectrum can be decomposed into three peaks at 533.3 eV, 531.9 eV, and 531.0 eV, corresponding to C=O, C-O, and O-N, respectively, based on the bonding interactions between carbon (C), nitrogen (N), and oxygen (O)[50]. The high-resolution N 1s spectrum (**Figure S8d**) was fitted with four components at 399.4 eV, 400.3 eV, 401.7 eV, and 402.7 eV, corresponding to Ti-O-N, -NH-, -N+=, and -N+H-, respectively. This increase in Ti-O-N content may result from the reaction between the -NH_2 group of polyaniline (PANI) and the -COOH group of $Ti_3C_2T_x$[48,49].

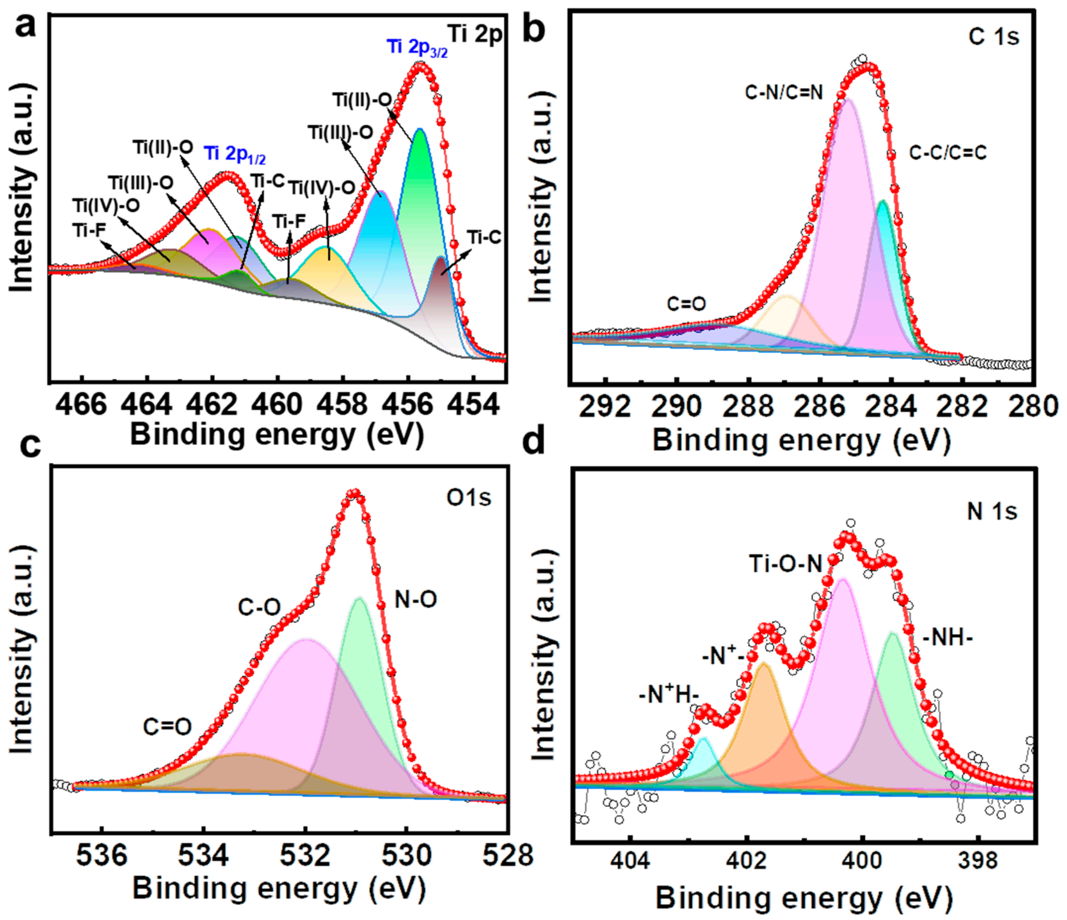

Figure S8. High-resolution XPS spectra of (a) Ti 2p, (b) C 1s, (c) O 1s, and (d) N 1s for $Ti_3C_2T_x$/PANI.

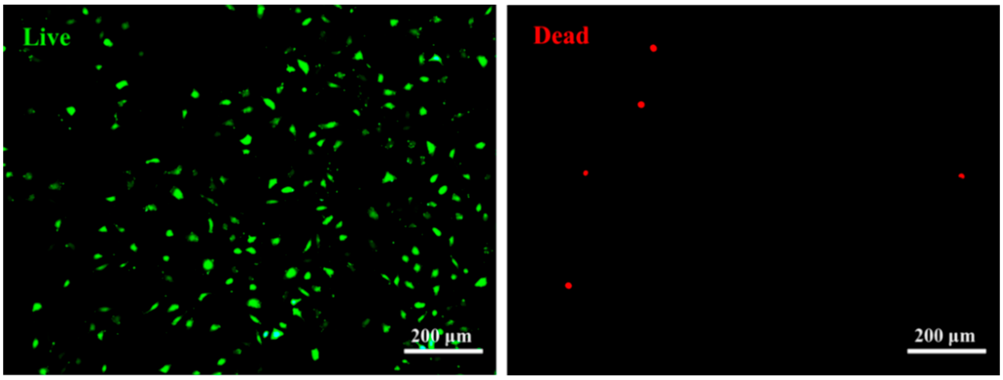

Figure S9. Fluorescence images of live and dead cells after cell viability test using CCK-8 assay.

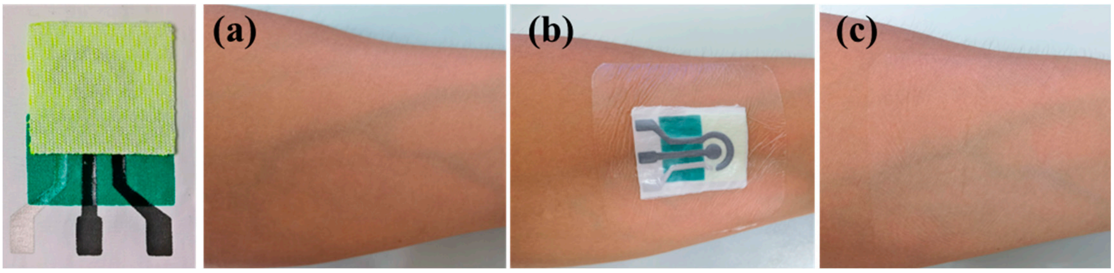

Figure S10. Photographs of the forearm taken before (a), during (b), and after (c) 24 hours of wearing the Janus textile integrated with the $Ti_3C_2T_x$ MXene/polyaniline-modified nylon fabric electrode, affixed using medical adhesive tape.

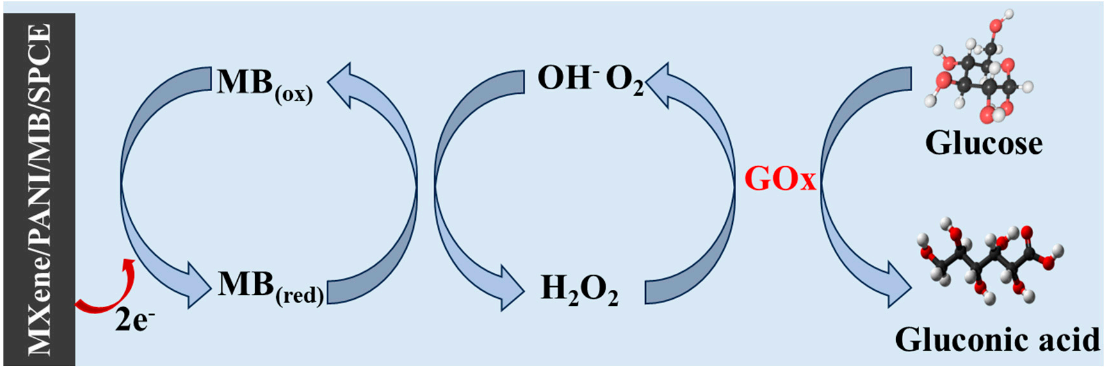

Figure S11. Schematic diagram showing the working mechanisms of glucose sensor.

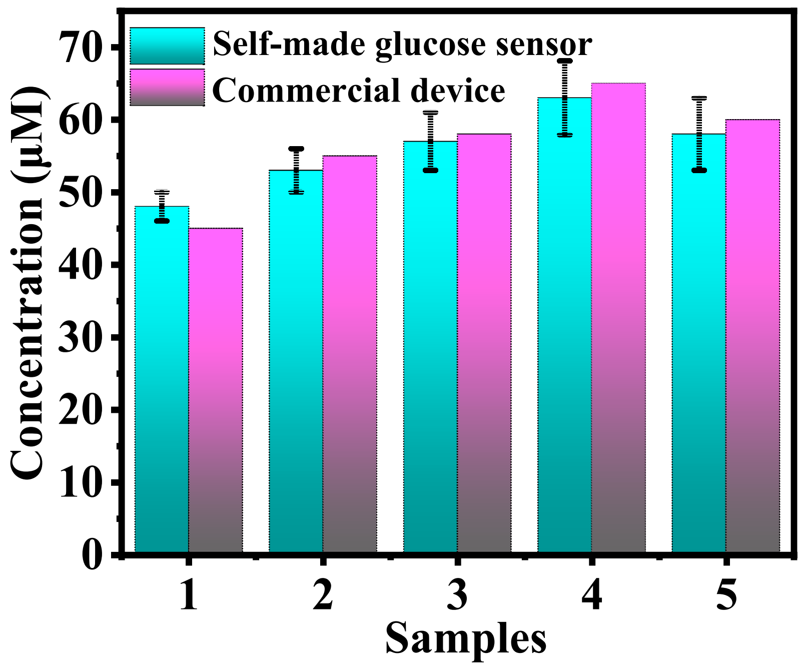

Figure S12. Detection of glucose concentration in sweat samples by a self-made glucose sensor and a commercial device, respectively.

As shown in Figure S13, bending durability was evaluated by subjecting the flexible electrode to repeated bending on cylindrical supports with radii of approximately 10 mm, 15 mm, and 18 mm. A representative configuration at a curvature radius of 10 mm is shown, where the electrode was subjected to 100 manual bending cycles, with each cycle defined as one complete bending and unbending motion. Electrochemical performance, particularly the glucose-induced current response, was assessed every 10 cycles. CV measurements were conducted at each bending radius, and the oxidation peak current (I_p) was extracted to assess the impact of mechanical deformation on electrochemical performance. Electrochemical measurements taken

before and after the 100 bending cycles revealed minimal signal degradation, indicating that the electrode maintained excellent structural integrity and electrochemical stability under repeated mechanical stress.

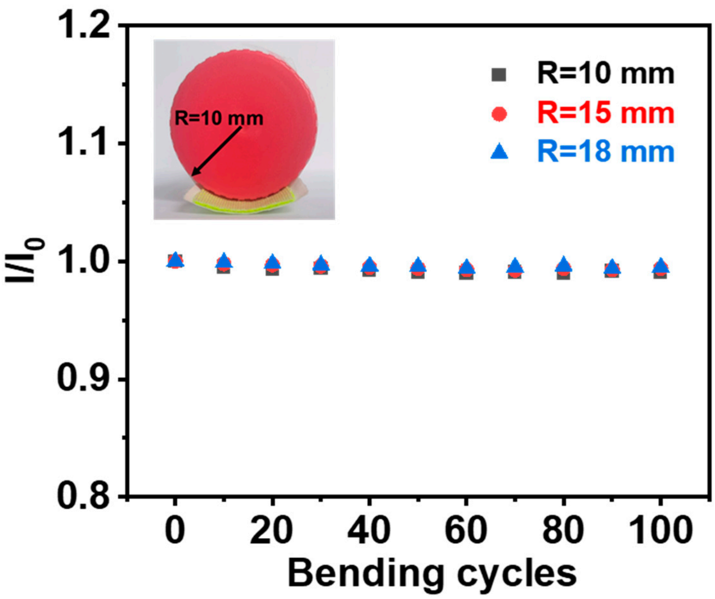

Figure 13. The ratio of the current (I/I_0) obtained from CV signals of the nylon-based sweat glucose electrochemical sensor after 100 bending cycles under various bending radii, where I and I_0 represent the peak oxidation current after and before bending, respectively.

As shown in Figure S14, a systematic amperometric evaluation was conducted over a 10-day period to assess the long-term stability of the sensor in a simulated sweat environment. During this period, the sensor maintained a stability rate exceeding 90%, with a relative standard deviation (RSD) of 3.29%, indicating that the fabricated working electrode exhibits good stability under physiologically relevant conditions.

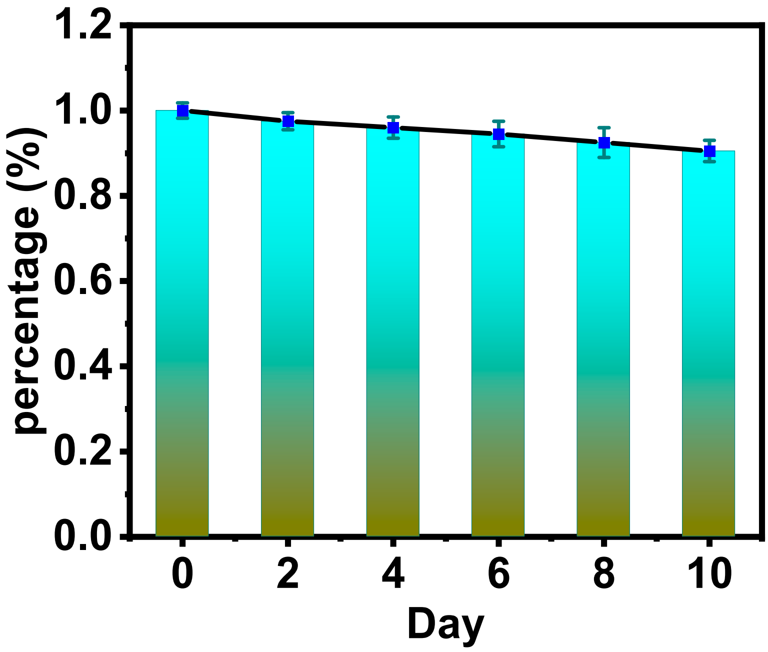

Figure S14. Storage stability of the biosensor in artificial sweat ([glucose] —100 μM).

Table S1. Comparison of the performance of the fabricated screen-printed electrochemical glucose sensor with other reported ones.

Electrode materials	Linear-range (mM)	Sensitivity ($\mu AmM^{-1}cm^{-2}$)	LOD (μM)	Potential (V)	Ref.
PPy/PB/GOx-modified graphite	0.05-0.5	1.9	/	0.05	[51]
Pt-Ir-alloy-PEEK	0.11-21	2.55	0.11	0.60	[52]
GOx/NiHCF/PB/Au/PET	0-0.2	0.0418	10	0.05	[53]
GOx/CVD graphene/Au mesh	0.01-0.7	1	10	−0.05	[54]
GOx/PB/MWCNT-Fe_3O_4/SPCE	0.001-0.4	4.495	0.38	0.23	[55]
GOx@PPtNPs@MWCNTs/PEDOT@PB	0.0025–1.250	21.6	2.5	-0.1	[56]
GOx/Pt-SilkNCT	0.025-0.3	0.0063	5	-0.2	[57]
GOx/Pt/rGO/ P3ABA	0.25-6	22.01	44.3	0.5	[58]
ZnO/MXene/GOx	/	27.87	17	−0.21	[59]
PEDOT:PSS/MXene/GOx	0.094-1.294	8.3	1.9	-0.375	[60]
Nafion/CHI-GOx/PB CBNPs	0.005-1.25	14.64	4.83	-0.05	[61]
Au/PET	0.02-1.11	22.05	2.7	0.1	[62]
Au/PB/GOx/CTS/GS	0.008-1	1.79	2.45	0.075	[63]
GOx/GTA/BSA/glycerol/Au	0.025-2	1.76	55	0.7	[64]
GOx/GO-Pt black/Pt	0.001-2	0.466	1	0.5	[65]
GOx/MB/PANI/$Ti_3C_2T_x$	0.04-3.08	3.11	4.82	-0.18	This work

References

[44] X. Wu, S. Wu, C. Wu, X. Zhang, Z. Jiang, S. Liu, N. Li, Plasma-promoted surface regulation of a novel integrative carbon network for boosting the long-cycle capability of sodium-ion storage, Carbon, 191 (2022), 112-121.

[45] F. Yuan, Y. Lei, H. Wang, X. Li, J. Hu, Y. Wei, D. Zhai, Pseudo-capacitance reinforced modified graphite for fast-charging potassium-ion batteries. Carbon, 185 (2021), 48-56.

[46] R. Liu, J. Li, M. Li, Q. Zhang, G. Shi, Y. Li, C. Hou, H. Wang, MXene-coated air-permeable pressure-sensing fabric for smart wear, ACS Appl. Mater. Interfaces 12 (2020) 46446-46454.

[47] C. He, Y. Tan, Y. Li, Conducting polyaniline nanofiber networks prepared by the doping induction of camphor sulfonic acid, J. Appl. Polym. Sci. 87 (2003) 1537-1540.

[48] J. Fu, J. Yun, S. Wu, L. Li, L. Yu, K. Kim, Architecturally robust graphene-encapsulated MXene Ti_2CT_x@Polyaniline composite for high-performance pouch-type asymmetric supercapacitor, ACS Appl. Mater. Interfaces 10 (2018) 34212-34221.

[49] P. Liao, Z. Geng, X. Zhang, W. Yan, Z. Qiu, H. Xu, High-performance $Ti_3C_2T_x$ achieved by polyaniline intercalation and

gelatinization as a high-energy cathode for zinc-ion capacitor, Nano Res. 17 (2024) 5305-5316.

[50] Z. Hou, H. Jiang, Y. Guo, K. Huang, F. Zhao, Y. Xu, J. Zhang, Enhancing acidic hydrogen evolution through pyrrolic nitrogen-doped reduced graphene oxide triggering two-electron oxygen reduction. Inorg. Chem. Front. 11 (2024), 4318-4328.

[51] A. Ramanavicius, A. Rekertaitė, R. Valiūnas, A. Valiūnienė, Single-step procedure for the modification of graphite electrode by composite layer based on polypyrrole, Prussian blue and glucose oxidase, Sens. Actuators B Chem. 240 (2017) 220-223.

[52] J. Li, P. Koinkar, Y. Fuchiwaki, M. Yasuzawa, A fine pointed glucose oxidase immobilized electrode for low-invasive amperometric glucose monitoring, Biosens. Bioelectron. 86 (2016) 90-94.

[53] Y. Lin, M. Bariya, H. Nyein, L. Kivimäki, S. Uusitalo, E. Jansson, W. Ji, Z. Yuan, T. Happonen, C. Liedert, J. Hiltunen, Z. Fan, A. Javey, Porous enzymatic membrane for nanotextured glucose sweat sensors with high stability toward reliable noninvasive health monitoring, Adv. Funct. Mater. 29 (2019) 1902521.

[54] H. Lee, T. K. Choi, Y. B. Lee, H. R. Cho, R. Ghaffari, L. Wang, H. J. Choi, T. D. Chung, N. Liu, T. Hyeon, S. H. Choi, D. H. Kim, A graphene-

based electrochemical device with thermoresponsive microneedles for diabetes monitoring and therapy, Nat. Nanotechnol. 11 (2016) 566-572.

[55] C. Shamili, A. S. Pillai, S. Saisree, A. Chandran, M. R. Varma, S. K. Peethambharan, All-printed wearable biosensor based on MWCNT-iron oxide nanocomposite ink for physiological level detection of glucose in human sweat, Biosens. Bioelectron. 258 (2024) 116358.

[56] S. Khumngern, N. Nontipichet, P. Thavarungkul, P. Kanatharana, A. Numnuam, Smartphone-enabled flow injection amperometric glucose monitoring based on a screen-printed carbon electrode modified with PEDOT@PB and a GOx@PPtNPs@MWCNTs nanocomposite, Talanta 277 (2024) 126336.

[57] W. He, C. Wang, H. Wang, M. Jian, W. Lu, X. Liang, X. Zhang, F. Yang, Y. Zhang, Integrated textile sensor patch for real-time and multiplex sweat analysis, Sci. Adv. 5 (2019) eaax0649.

[58] S. Phetsang, J. Jakmunee, P. Mungkornasawakul, R. Laocharoensuk, K. Ounnunkad, Sensitive amperometric biosensors for detection of glucose and cholesterol using a platinum/reduced graphene oxide/poly(3-aminobenzoic acid) film-modified screen-printed carbon electrode, Bioelectrochemistry 127 (2019) 125-135.

[59] V. Myndrul, E. Coy, N. Babayevska, V. Zahorodna, V. Balitskyi, I. Baginskiy, O. Gogotsi, M. Bechelany, M.T. Giardi, I. Iatsunskyi, MXene nanoflakes decorating ZnO tetrapods for enhanced performance of skin-attachable stretchable enzymatic electrochemical glucose sensor, Biosens. Bioelectron. 207 (2022) 114141.

[60] Y. Pan, M. He, J. Wu, H. Qi, Y. Cheng, One-step synthesis of MXene-functionalized PEDOT: PSS conductive polymer hydrogels for wearable and noninvasive monitoring of sweat glucose, Sens. Actuators B Chem. 401 (2024) 135055.

[61] C. Tang, K. Zhou, R. Wang, M. Li, W. Liu, C. Li, X. Chen, Q. Lu, Y. Chang, Wearable biosensors for human sweat glucose detection based on carbon black nanoparticles, Anal. Bioanal. Chem. 416 (2024) 1407-1415.

[62] Y. Wang, X. Wang, W. Lu, Q. Yuan, Y. Zheng, B. Yao, A thin film polyethylene terephthalate (PET) electrochemical sensor for detection of glucose in sweat, Talanta 198 (2019) 86-92.

[63] B. Li, X. Wu, C. Shi, Y. Dai, J. Zhang, W. Liu, C. Wu, Y. Zhang, X. Huang, W. Zeng, Flexible enzymatic biosensor based on graphene sponge for glucose detection in human sweat, Surf. Interfaces 36 (2023) 102525.

[64] A. Müsse, F. La Malfa, V. Brunetti, F. Rizzi, M. De Vittorio, Flexible enzymatic glucose electrochemical sensor based on polystyrene-gold electrodes, Micromachines 12 (2021) 805.

[65] J. Shi, H. Zhang, A. Snyder, M. Wang, J. Xie, D. Marshall Porterfield, L. A. Stanciu, An aqueous media-based approach for the preparation of a biosensor platform composed of graphene oxide and Pt-black, Biosens. Bioelectron. 38 (2012) 314-320.
